# Response to Fraser & Wark’s comments on *A new theory for X-ray diffraction*

**DOI:** 10.1107/S2053273318007489

**Published:** 2018-07-18

**Authors:** Paul F. Fewster

**Affiliations:** a Brighton, UK

**Keywords:** diffraction theory, powder diffraction, small crystals

## Abstract

The criticisms of my theory, as given by Fraser & Wark [(2018), *Acta Cryst.* A**74**, 447–456], are built on a misunderstanding of the concept and the methodology I have used. The assumption they have made rules out my description from which they conclude that my theory is proved to be wrong. They assume that I have misunderstood the diffraction associated with the shape of a crystal and my calculation is only relevant to a parallelepiped and even that I have got wrong. It only appears wrong to Fraser & Wark because the effect I predict has nothing to do with the crystal shape. The effect though can be measured as well as the crystal shape effects. This response describes my reasoning behind the theory, how it can be related to the Ewald sphere construction, and the build-up of the full diffraction pattern from all the scatterers in a stack of planes. It is the latter point that makes the Fraser & Wark analysis incomplete. The description given in this article describes my approach much more precisely with reference to the Ewald sphere construction. Several experiments are described that directly measure the predictions of the new theory, which are explained with reference to the Ewald sphere description. In its simplest terms the new theory can be considered as giving a thickness to the Ewald sphere surface, whereas in the conventional theory it has no thickness. Any thickness immediately informs us that the scattering from a peak at the Bragg angle does not have to be in the Bragg condition to be observed. I believe the conventional theory is a very good approximation, but as soon as it is tested with careful experiments it is shown to be incomplete. The new theory puts forward the idea that there is persistent intensity at the Bragg scattering angle outside the Bragg condition. This intensity is weak (∼10^−5^) but can be observed in careful laboratory experiments, despite being on the limit of observation, yet it has a profound impact on how we should interpret diffraction patterns.

## Abstract 2

In response to the comments by Fraser & Wark [(2018), *Acta Cryst.* A**74**, 447–456], experimental evidence and an explanation of the new theory in the context of a modified Ewald sphere construction are presented.

## 1. Introduction   

The new theory of X-ray diffraction arose from trying to account for inexplicable experimental observations. Neither the conventional dynamical nor kinematical theories could explain the measurements. The microstructure would have to be fantastical to account for some of these observations. Several experimental examples are included in this article that support the theoretical interpretation. My questioning of conventional theory started in the 1990s when using the near-perfect diffraction space probe (Fewster, 1989 ▸) to study polycrystalline materials and perfect semiconductors, with work on a different description beginning in the mid-2000s. It was clear that the observed features could no longer be dismissed as artefacts of the instrument, requiring an alternative explanation of experimental data.

This article is in five sections. The first relates the new theory to the Ewald sphere construction to give a better visual description, which is achieved by simply translating equation (5) of Fewster (2014 ▸) into graphical form. The second part describes the build-up of the scattering and where the intensity is concentrated, including the simple error/misunderstanding/assumption made by Fraser & Wark (2018 ▸). The third section gives some experimental evidence of the persistent intensity at the Bragg scattering angle when not in the Bragg condition. The fourth section considers the impact of crystal shape. The fifth section lists some of the examples that are difficult to explain using the conventional theory that are easily explained with the new theory.

## 2. The relationship of the new theory to the Ewald sphere   

**Figure 1:**
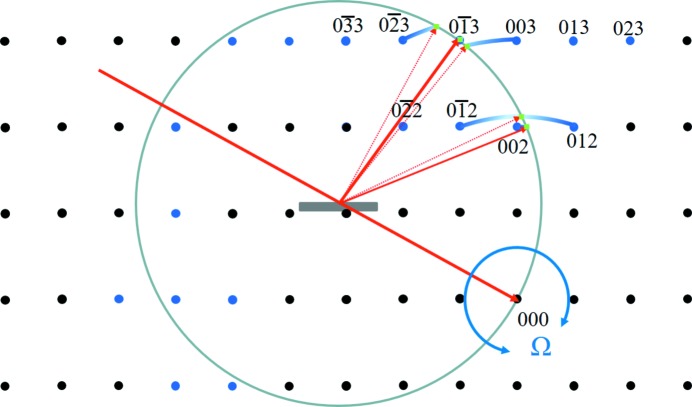
The new theory in terms of the Ewald sphere construction. All the reciprocal-lattice points coloured blue can form intensity at this incident angle at their respective 2θ_B_ values (*e.g.* green dots) if 0 < Ω < 2θ_B_. The distance of the reciprocal-lattice point to the surface of the Ewald sphere along an arc in Ω defines its amplitude, which decreases as the distance increases. For example, 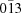 is in the Bragg condition and the amplitude is at its maximum value, whereas 002 is weaker and 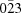 is very weak *etc.* The arcs drawn for some of the reflections give a guide to the strength of the scattering. The Ewald sphere surface can be considered to have a thickness with a profile given by equation (4) of Fewster (2014 ▸).

The whole basis of the new theory is that a strong scattering feature, *e.g.* a Bragg peak, can still be observed as the crystal is rotated away from its position on the Ewald sphere. This applies to all the diffraction features, *e.g.* thickness fringes and crystal truncation rods, but will be weak. The distance of a diffraction feature from this ‘conventional’ Ewald sphere surface is given by the length of the arc of a vector (for the feature of interest) rotated about 000 (Fig. 1 ▸). The length of the vectors in the figure corresponds to 1/*d_hkl_*. The arcs touch the Ewald sphere at 2θ*_hkl_* with a residual amplitude given by equation (4) of Fewster (2014 ▸). The next section explains why there is intensity at this position. Thus, a considerable proportion of the full diffraction pattern should be observed if there is sufficient intensity. This is exactly what we would expect from optical diffraction. Rotating the crystal just increases or decreases the intensity of the features in the diffraction pattern, *e.g.* Bragg peaks, thickness fringes, crystal truncation rods, fringes from spherical crystals *etc.*, and when they coincide with the surface of the ‘conventional’ Ewald sphere the intensity for that feature reaches its maximum value. The ‘conventional’ Ewald sphere just represents the specular condition and has no width. The new theory just expresses that there is a residual specular contribution that does not go to zero as soon as the feature giving rise to it is rotated away from the optimum position on the sphere surface.

There is also a philosophical question here: if the Ewald sphere has no width then how can a reciprocal-lattice point interact with it? If the crystal is stationary, the source is monochromatic and there is no beam divergence, what would the intensity be? This was a serious problem for Wojtas *et al.* (2017 ▸) in their interpretation of XFEL (X-ray free-electron laser) data, requiring the partial capture of a reciprocal-lattice point and invoking angular tolerances to obtain some explanation of the data. If there were too many ‘Bragg peaks’ then they assumed that they were capturing data from more than one crystal and rejected the data. The new theory defines a width for the sphere surface and this dilemma does not exist. Because it has a width then intensity will be captured away from the Bragg condition. The new theory describes the thickness profile and the associated residual amplitude that is captured.

**Figure 2:**
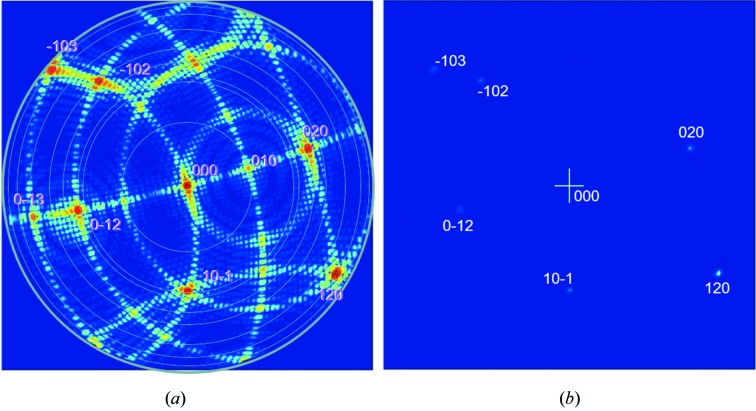
The simulation of the diffraction pattern from a three-dimensional array of point scatterers with dimensions 40 × 39 × 40 nm with point separations of 2 × 3 × 4 nm using a wavelength of 1.54 nm. The whole pattern is revealed in a logarithmic plot (*a*). When plotted on a linear scale (*b*) there are six ‘peaks’ observed. This is very characteristic of data from XFELs. Diffraction based on the conventional theory would reveal nothing in this arbitrary orientation (these are not in the Bragg condition). The central peak in (*a*) is the direct beam and is removed from the linear plot in (*b*), to reveal the other peaks with linear scaling. The plots are displayed on a radius of 2θ out to a maximum of 90°. The peaks can be indexed based on their 2θ_B_ values and the restriction 0 < Ω < 2θ_B_, yet their intensities vary significantly indicating that the reciprocal-lattice points cannot all be close to their Bragg conditions. It can be seen in (*b*) on a linear scale that peak intensities <∼1% of the most intense peak are not observed.

So, what evidence is there for this? Well there is plenty of evidence, from calculating the diffraction pattern from first principles, results from XFEL sources and even data collected from standard laboratory sources. Let us start with the calculated evidence from my colleague John Anderson and presented by Fewster (2017 ▸). This considers a single-wavelength plane wave impinging on a three-dimensional array of point scatterers, which will form a spherical wave from each point. When the scattering is brought together in the far field, *i.e.* the waves travelling in a parallel scattered direction are brought together, a diffraction pattern is formed. The phases of the contributions depend on the difference in path lengths of all the contributions at each 2θ value. The first thing to notice is that the full diffraction pattern exists (Fig. 2 ▸ *a*). That is not predicted in conventional theory where intensity from a feature only occurs when it touches the surface of the Ewald sphere. This figure is plotted on a logarithmic scale to reveal the detail. For a real experiment the data will have a finite dynamic range and only the strong features are likely to be observed (Fig. 2 ▸ *b*). These simulations reveal the fringing due to the crystal surface boundary conditions (the shape transform) and if a fringe is close to the Ewald sphere then it could be more intense than the associated Bragg peak that is more remote, *e.g.* Fewster (2016 ▸) and Fig. 5 below. These calculations do not contain any complicated parameters (wavelength dispersion or divergence *etc*.), yet the resulting diffraction patterns are very similar to those observed at XFELs, *i.e.* several peaks in an instantaneous image, occasional row of fringes *etc.*, depending on where the dynamic range of these calculations is truncated. The diffraction pattern can be indexed from the 2θ_B_ of the observed peaks.^1^

Studying these images in greater detail and concentrating on the 2θ_B_ positions for the Bragg peaks, it is possible to observe intensity enhancement at these angles for this single incident angle. It must be recognized though that there will be peak movements resulting from the interference of the amplitude oscillations related to shape effects and those related to the enhancement effect as Ω is varied. This will also be influenced by how close their contributions are to the surface of the Ewald sphere. The overlap of fringes from reflections of different order will also influence the observed diffraction pattern, which is particularly relevant for small, perfect crystals (Holý & Fewster, 2008 ▸; Fewster, 2015 ▸, 2018 ▸). We can separate out the shape effects by extending the familiar description of Bragg’s law.

## 3. The explanation of the persistent peak at 2θ_B_ and response to the Fraser & Wark analysis   

**Figure 3:**
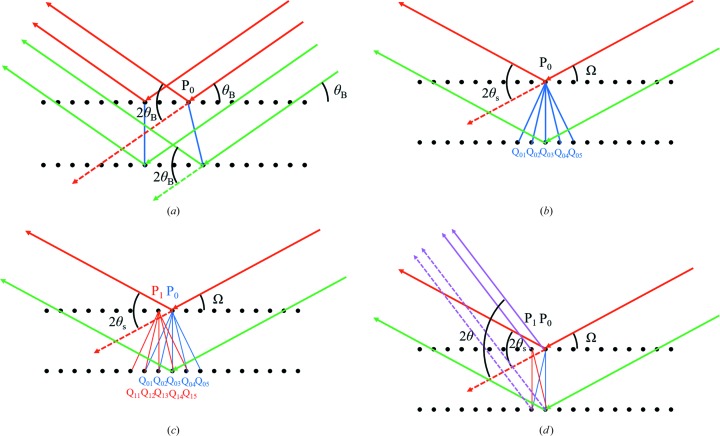
(*a*) The Bragg condition, where all the scattering from all the positions on both planes is in phase, so any pairing of a scattering point from one plane with any point on another plane will be in phase. (*b*) When the scattering planes are rotated away from the Bragg angle a point P_0_ cannot scatter in phase with any point Q at the specular scattering angle 2θ_s_. (*c*) For the same incident angle and the same specular scattering angle the near-phase relationship holds across the plane for P_0_, P_1_ *etc*. (*d*) However, if we move the detector to a different 2θ, P_0_ and P_1_ no longer scatter perfectly in phase and similarly the phase relationship associated with P and Q points will change. The phase relationship between the scattering from P and Q points can therefore be varied by moving the detector. If there is a detector position where the path length difference is λ then all the planes will scatter in phase, with a maximum value defined by the phase sum of the amplitudes of points P_0_, P_1_ *etc*.

A series of diagrams (Fig. 3 ▸) is given that explains the thinking behind the new theory and the reasoning of Fraser & Wark to make it clear where their misunderstanding has occurred.

A point P_0_ on the upper plane will be in phase with any point in any position on the lower plane Q when in the Bragg condition, which in turn will also be in phase with all other points on the upper plane (Fig. 3 ▸ *a*). When the planes are rotated away from the Bragg condition, the point P_0_ will have a close phase relationship with several points on the lower plane, Q_01_, Q_02_, Q_03_, Q_04_ *etc*., and we would expect to see some residual intensity at the specular angle (Fig. 3 ▸ *b*). The point P_0_ can never be exactly in phase with a Q_0_ point for this combination of Ω and 2θ outside the Bragg condition (*i.e.* Ω = θ_B_). The Fraser & Wark analysis to this point would be the same; then they consider this angular spread of acceptable phases combined with the density of scattering points on the lower plane to give rise to an intensity. I have no dispute with this.

If we now include another point on the top plane, which we call P_1_ (Fig. 3 ▸ *c*), then there will be another set of points on the lower plane that have the same relationship as for P_0_. We shall call these points Q_11_, Q_12_, Q_13_, Q_14_ *etc*. These scattering points on the lower plane Q_1*n*_ will have some overlap with the points Q_0*n*_. Since there are as many scattering points on the P and Q planes we should pair every P point with a Q point, and the conclusion would be the same as before if all the P points are in phase (Fig. 3 ▸ *c*). This arrangement of scattering points produces a peak of intensity at the specular scattering angle that we can call 2θ_s_. This scattering angle is defined by the crystal surface where the scattered wave exits the crystal and is a result of the boundary condition, which requires the component of the electric field parallel to the surface of the crystal to be continuous. This explains the fringing associated with the crystal shape, often termed the shape transform. If the incident angle is not equal to the Bragg angle, then 2θ_s_ can never equal 2θ_B_. This is the conclusion in Fraser & Wark that I agree with; it is purely a conclusion of the conventional theory.

What happens if the detector is moved to a different 2θ angle, whilst maintaining the same incident angle? The description of Fraser & Wark or the conventional theory does not consider this. The scattering does not correspond to the specular condition (Fig. 3 ▸ *d*) and P_0_ is no longer in phase with P_1_ and similarly the phase relationship between the scattering from the points P and Q has changed. Conventional theory and that of Fraser & Wark simply assume that intensity only exists when the points P are perfectly in phase. But what happens if the points P scatter slightly out of phase? Is it realistic to assume that there is no intensity in this case? This is a major anomaly in the conventional theory and can be interpreted as the Ewald sphere surface having no thickness.

**Figure 4:**
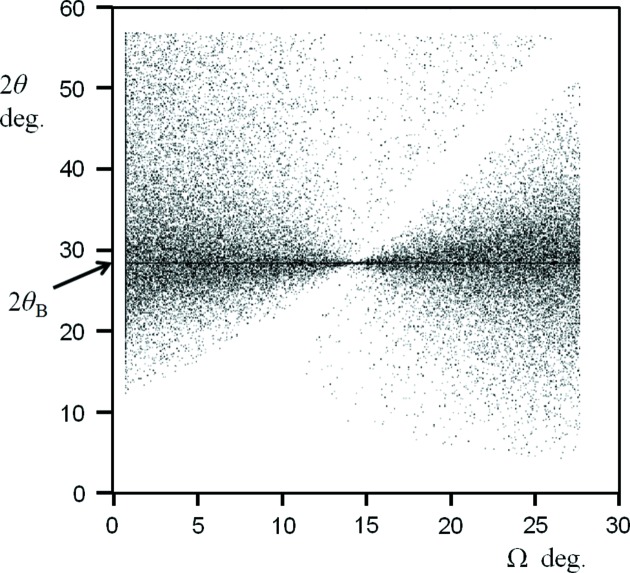
The distribution of path lengths equal to one wavelength (to within a very small tolerance) from scattering points on adjacent planes. As the tolerance is reduced it concentrates on a single value at 2θ_B_ and the other coincidences become sparser.

If we postulate that the points P_0_ and P_1_ can scatter in a less than perfect phase alignment, then we must conclude that there is intensity outside the specular condition. This has nothing to do with crystal shape. If the detector is moved further the phase relationships between all the P points and all the Q points will change again. Because the phase relationship between all P points can be determined and every P to every Q can be determined, the PQ pair can be paired in an arbitrary way. It is convenient to find the PQ pair that forms a path length difference closest to one wavelength. The phase difference between P_0_, P_1_, P_2_ *etc.* is determined purely by the incident angle Ω to their plane and the detection point 2θ [equation (4), Fewster (2014 ▸)], which defines the maximum amplitude possible from the P plane for this Ω at 2θ, *i.e. A*_Ω_. *A*_Ω_ applies to the second and all subsequent planes and the maximum amplitude that can exist for this incident angle occurs when all planes scatter in phase with each other, *i.e. NA*_Ω_ where *N* is the number of planes. This will only occur if there are PQ pairings that have a path length of one wavelength. By taking a point P on the upper plane and an incident angle Ω, we search for a pairing with a Q position that will give a path length difference of one wavelength by allowing 2θ to take on any value. Fig. 4 ▸ is a plot of the angle combinations Ω and 2θ where a one-wavelength path difference can exist between a P position and a Q position. For any given incident angle Ω there is a one-wavelength path difference possible at 2θ_B_. We can consider that an incident angle below the Bragg angle will form a specular peak at 2θ_s_ with a maximum path length difference < λ and by increasing 2θ the path length difference can be increased. Similarly, for an incident angle above the Bragg angle a specular peak will form at 2θ_s_ with a minimum path length difference > λ and by reducing 2θ the path length difference can be decreased. In both cases we can achieve a path length of λ to form an amplitude of *NA*_Ω_.

This same analysis can be performed for any part of the truncation rod; however, the path length difference never reaches one wavelength but would be associated with a path length above or below this value. The conclusion is that the diffraction pattern is rich with information as in Fig. 2 ▸(*a*). This approach ensures that all scattering centres across these planes and by extension all planes in the stack are included.

The new theory therefore predicts that a scan in 2θ over a large range at a fixed incident angle would encounter a peak at 2θ_s_ corresponding to the specular condition (*e.g.* crystal truncation rod) and at 2θ_B_ (the enhancement or persistent peak). This is exactly what was observed by Fewster (2016 ▸) and further clearer examples are given in the following section, including the measurement of the predicted arc in Fig. 1 ▸ [example (iv) in §4].

## 4. Experimental evidence from laboratory sources   

**Figure 5:**
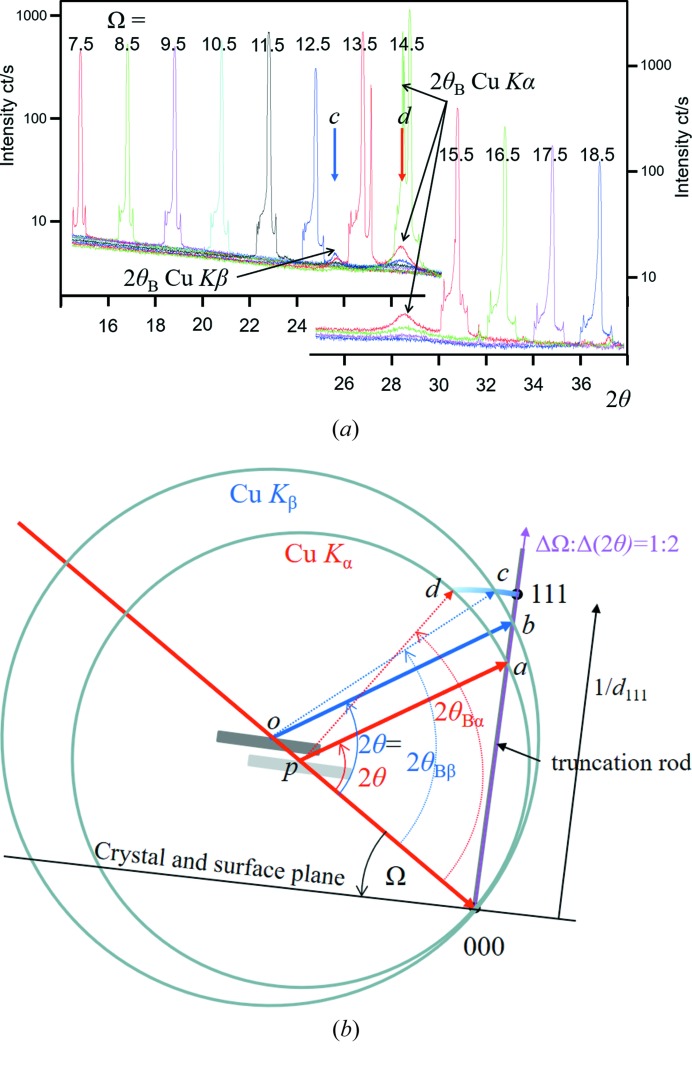
(*a*) Several 2θ scans for fixed Ω settings with the interpretation in (*b*) based on the modified Ewald sphere construction. The spheres have different radii: 1/λ_α_ and 1/λ_β_, centred on *p* and *o*, respectively. Consider the 2θ scan for Ω = 12.5° in (*a*) (the crystal is orientated 1.7° from the Bragg angle θ_Bα_ for the Cu *K*α wavelength). There is a single specular peak (the intersection of the 2θ scan and the truncation rod) that is described in (*b*), where the specular contributions occur at the same 2θ but capture different positions on the truncation rod at *a* and *b*, which is the same for both conventional and new theories. The two peaks, *c* and *d*, correspond to the *d*_111_ plane spacing for both the Cu *K*α and Cu *K*β wavelengths, *i.e.* 2θ_α_ and 2θ_β_; in the conventional description these should not exist. The peaks at *c* and *d* can only be described with the new theory, *i.e.* the persistent intensity at 2θ_α_ and 2θ_β_. The 2θ_α_ peak can be observed up to |Ω − θ_B_| ∼ 6°. The specular peaks are sharp (they are dominated by the proportion of the incident-beam divergence that satisfies this condition, *i.e.* a small region on the sample), and the enhancement peaks are broad (because all the incident-beam divergence directions will form intensity at 2θ_B_ and these exist over the full footprint of the beam on the sample. As the Bragg condition is approached the peak will sharpen because the strongest contributions come from a smaller range of divergence and smaller regions on the sample and dominate). The features at the base of the specular peaks are tube focus artefacts.

(i) The first example was an early test of my theory. The sample is a large, perfect crystal wafer of 111-oriented silicon. The incident beam (Cu *K*) is collimated to give an angular divergence of 0.03° and the crystal is set to several incident angles, Ω, either side of the 111 Bragg angle (θ_B_). The scattering is captured by scanning in 2θ (Fig. 5 ▸ *a*). Peaks are observed that correspond to the intersection of the crystal truncation rod at 2θ = 2Ω and further peaks at 2θ = 2θ_B_ for both the Cu *K*α and Cu *K*β wavelengths for the *d*_111_ crystal planes. The 2θ = 2θ_B_ peaks are observed for incident angles up to 6° away from the Bragg condition.

(*a*) How can a crystal set at an incident angle remote from the Bragg condition produce a peak at 2θ_B_?

(*b*) How can two 2θ_B_ peaks associated with different wavelengths that require different incident angles be observed simultaneously?

**Figure 6:**
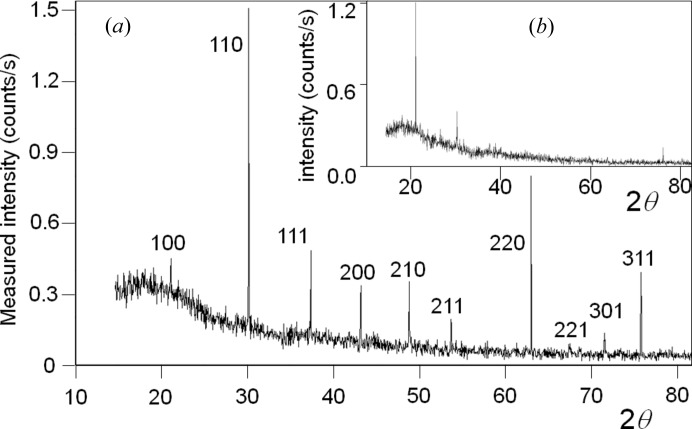
[Fig. 3 from Fewster (2014 ▸)]: (*a*) the scattering pattern from ∼120 crystals (or if perfectly packed 300 crystals) isolated with a 3.5 µm incident beam that perpendicularly intersects a 1 mm-wide single layer of crystals of LaB_6_ with sizes of 2 to 5 µm. (*b*) gives the profile with ∼30 crystallites or if perfectly packed 75 crystallites (3.5 µm × 0.25 mm sample size), where only three reflections are clearly resolved compared with all ten in the larger sample size. The data were collected with a 0.01° divergent Cu *K*α_1_ beam from a 1.8 kW X-ray laboratory source in 35 min. The samples were stationary throughout, so the incident beam only explored one orientation from each crystal. The peaks are narrow and occur at the correct 2θ_B_ positions and correspond to the interpretation where each crystal contributes intensity as in Fig. 1 ▸.

The explanation based on the new theory is given in Fig. 5 ▸(*b*), and because of the large dimension parallel to the surface the shape function is dominated by the crystal truncation rod. The residual peaks at 2θ_B_ follow the prediction of equation (4), Fewster (2014 ▸).

(ii) A very highly collimated monochromatic beam 3.5 µm wide (horizontal with a divergence of 0.01°)^2^ is incident on a 1 mm-wide (vertical) polycrystalline sample to form a cross section of 0.0035 mm^2^ that is one crystal thick. The average crystal is 3.5 µm in diameter; this illuminated area and absorption measurements (to estimate the packing density) suggest there are ∼120 crystals being illuminated. The sample is kept stationary and the scattering is captured on a position-sensitive detector (the angular spread normal to the scattering plane is limited to 2.3° with a Soller slit). All ten possible peaks at their correct 2θ_B_ are observed and are sharp (Fig. 6 ▸ *a*). The probability of capturing one crystal in the Bragg condition is 1 in 23 000, and therefore to capture all ten is 1 in 4 × 10^43^.

(*a*) How, when the probability of observing a peak at the Bragg condition is 1 in 100 000, can a repeat experiment with ∼30 crystals form three clear peaks (Fig. 6 ▸ *b*)?

(*b*) Is it reasonable to expect each crystal to be composed of ∼100 000 mosaic blocks?

(*c*) If there are 100 000 mosaic blocks in each crystal, they would have an average diameter of ∼0.075 µm. How can the average intrinsic width for these mosaic blocks (∼0.11°) be reconciled with the measured width of 0.026°?^3^

**Figure 7:**
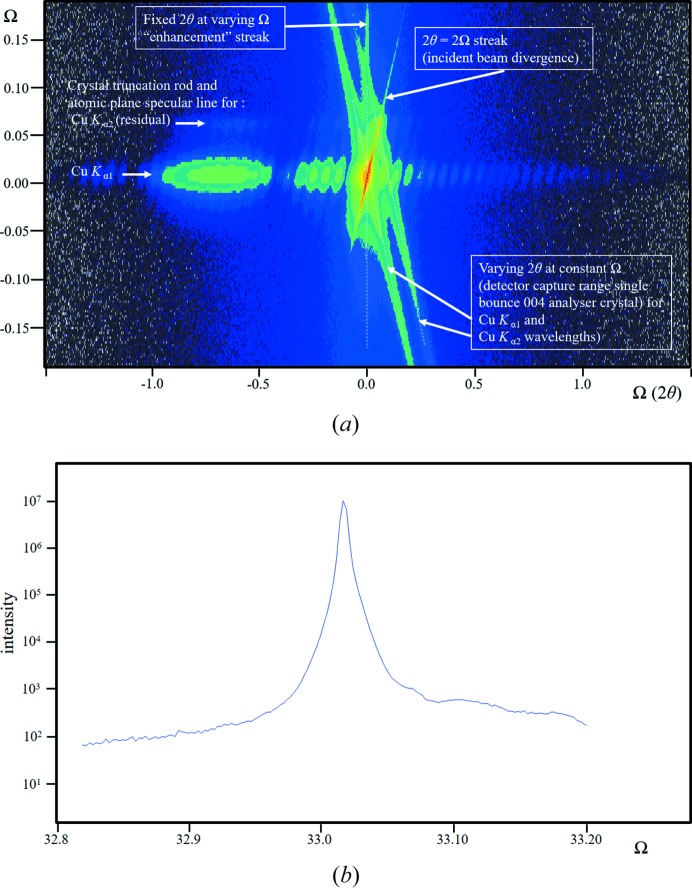
(*a*) A diffraction space map close to the 004 reflection (logarithmic scale) from an InGaAs structure grown epitaxially on a GaAs substrate. The data were collected with the beam selection diffractometer (Fewster, 2004 ▸), with a single reflection 004 analyser crystal (stepping in Ω followed by a scan with movements in Ω and 2θ maintaining a 1:2 ratio). The strong fringing is associated with the layer structure (the shape transform) and occurs along the crystal surface normal. The streak where 2θ = 2Ω corresponds to the incident-beam divergence and the streak along 2θ for a constant Ω value corresponds to the detector acceptance range (in this case the diffraction profile of the analyser crystal). The remaining streak at constant 2θ_B_ for varying Ω values is the ‘enhancement’ peak for the substrate (as in Fig. 5 ▸ *b*). (*b*) This is the extracted profile along the 2θ_B_ enhancement that is smoothly decreasing from the peak as expected, apart from interference of the Cu *K*α_2_ streak on the high-angle side. If all the artefacts could be removed and the alignment improved, this could be considered as the thickness profile of the Ewald sphere surface for this reflection and crystal.

The new theory has a simple explanation by building all the weak contributions from each crystal as in Fig. 1 ▸.

(iii) This is an example of the data from the beam selection diffractometer (Fewster, 2004 ▸). This instrument creates very high intensity, near-‘zero’ wavelength dispersion and well understood instrumental artefacts. The scattering from the sample 004 reflection is captured with a single reflection 004 analyser crystal (Fig. 7 ▸ *a*). The sample is a perfect crystal. The combination of the analyser crystal and a slit to control the wavelength dispersion still shows the remnants of the Cu *K*α_2_ component. In addition to the layer thickness fringes, there are the influences of the incident-beam divergence and the detector acceptance, which are clearly revealed as streaks emanating from the intense substrate peak. In addition, there is a prominent streak at constant 2θ_B_. The crystal plane rotation is not accurately normal to the reciprocal-lattice mesh, so this streak is inclined to the plane of the diffractometer.

(*a*) What is the explanation for the streak of intensity at constant 2θ_B_ as the crystal is rotated in Ω?

**Figure 8:**
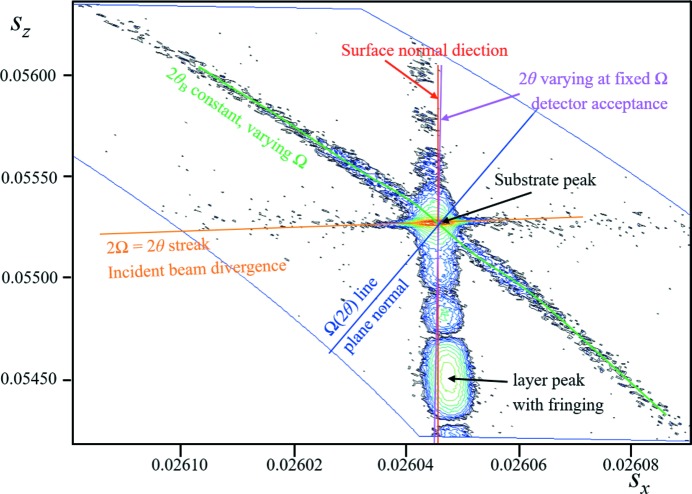
The complex scattering (logarithmic scale) close to the 113 reflection from a Si (001) wafer, with a 46 nm epitaxial layer of Si_0.21_Ge_0.79_ on top, obtained with a high-resolution diffractometer, courtesy of A. Kharchenko and J. Woitok. The fringing relates to the thickness of the SiGe layer, which can all be explained by conventional (dynamical) theory. The various features determined by the instrument and diffraction geometry are given in the figure and can be related to those in Fig. 7 ▸(*a*). The streak of intensity at constant 2θ_B_ cannot be explained with conventional theory but is predicted by the new theory and corresponds to an arc in Fig. 1 ▸.

The new theory predicts this 2θ_B_ streak, its shape and how it changes with crystal alignment. Fig. 7 ▸(*b*) gives an indication of the intensity along the 2θ_B_ streak for this sample, *i.e.* 10^−5^ to 10^−6^ of the Bragg peak at an angle of 0.15° from the Bragg condition.

(iv) This example uses a high-resolution monochromator and a position-sensitive detector to study a (001)-oriented Si wafer that has a single Si_0.21_Ge_0.79_ 46 nm layer grown epitaxially on top. The data were collected close to the 113 reflection by stepping in Ω and scanning in 2θ, and plotted in reciprocal-space coordinates forming an arc of captured data (Fig. 8 ▸). The SiGe layer is tilted with respect to the substrate,^4^ giving a tilted truncation rod (their individual crystal truncation rods are not coincident but still interfere with each other). The substrate gives rise to the most intense peak and the layer gives a broad peak with fringes. The influence of the incident-beam divergence and the 2θ capture line for a fixed incident angle can all be explained within the description of conventional theory. The substrate is perfect device-grade Si and is not mosaic. There is a very prominent arc of intensity at constant 2θ_B_ which corresponds exactly to the substrate *d*_113_ plane spacing. This is the persistent intensity or ‘enhancement’ predicted by the new theory.

(*a*) Is there any explanation within the confines of conventional theory that can explain this arc of intensity at constant 2θ_B_ from a perfect crystal as it is rotated in Ω?

This arc of intensity follows the description in Fig. 1 ▸ (and discussed later in Fig. 10). It cannot be described by any shape function.

**Figure 9:**
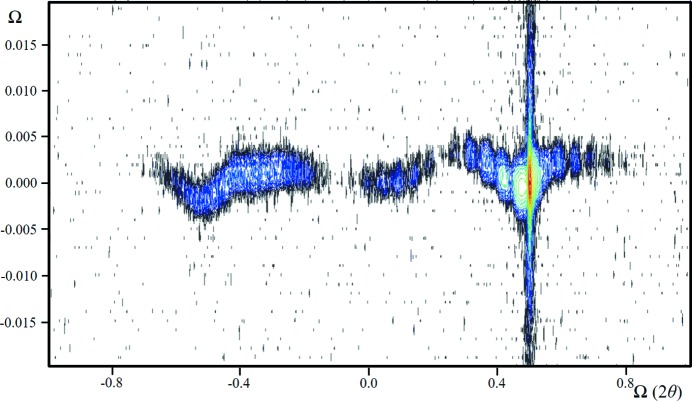
The 004 diffraction space map (logarithmic scale) expanded normal to the crystal truncation rod to emphasize the wavy streak of the 80 Å In_0.15_Ga_0.85_As quantum well, buried in a complex AlGaAs/GaAs structure. The other dominant feature is the streak along 2θ_B_. When the data were projected along 2θ_B_, the resultant profile fitted precisely with the simulation based on dynamical theory. Collecting data with a high-resolution diffractometer without an analyser (a rocking curve) gave small fringe displacements with a broadened base to the substrate peak (a commonly observed feature, which can be associated with the 2θ_B_ enhancement for varying Ω), whereas a single scan along the crystal truncation rod gave regions of missing intensity.

(v) This example is taken from a careful experiment on a structure composed of two epitaxial layers of GaAs/InGaAs on a GaAs substrate. The structure appears to be perfect until it is studied in greater detail with a very high resolution diffractometer (Fewster, 1989 ▸) (Fig. 9 ▸). There are two significant features that are observed: a crystal truncation rod that ‘wiggles’^5^ and an intensity streak along 2θ_B_ associated with the substrate. These features are a common observation in well aligned, good quality crystals, for layer structures and blank crystal wafers. The fringes associated with the layers indicate that the interfaces are flat and parallel. There is interference between the crystal truncation rod for the substrate and the layers, which is only possible if there is significant overlap. The intensity spreading at constant 2θ_B_ for each part of the structure would account for this overlap and the wiggles.

(*a*) How can the truncation rods of the substrate and layers interfere without some overlap to create these ‘wiggles’?

(*b*) What is the reason for the 2θ_B_ streak that also gives rise to a broadened base of the substrate peak in an open detector rocking curve?

The new theory predicts the existence of the streak in 2θ_B_, which in turn will account for the interference of the crystal truncation rods to explain the ‘wiggles’. It also indicates how a full two-dimensional diffraction space map can be simulated.

## 5. The impact of crystal shape   

The crystal shape will modify the intensity close to the Bragg peak, which was recognized by Fewster (2014 ▸) p. 262: ‘Hence a powder sample that has a distribution of orientations will create fringes associated with its size and surface shape and an enhancement at 2θ_B_ for each crystallite plane’. The main thrust of this theory is to concentrate on the persistent intensity at 2θ_B_, whereas all shape effects will modify the intensity around the Bragg condition peak and will not form intensity at 2θ_B_ unless by chance. Equation (5) in Fewster (2014 ▸) can be considered as the formula for a crystal wafer with crystal planes parallel to the surface. For other crystal shapes, the full shape transform can be included, but the position of the Bragg condition is unchanged. To include the shape transform for a parallelepiped, as in the work of James (1962 ▸) and Authier (2001 ▸), for a small crystal, would involve extra terms in equation (5), *i.e.* of the form 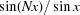 and 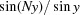. Since so few crystals conform to this shape I refer to my original statement above, *i.e.* any shape can be included but the persistent intensity at 2θ_B_ still exists.

**Figure 10:**
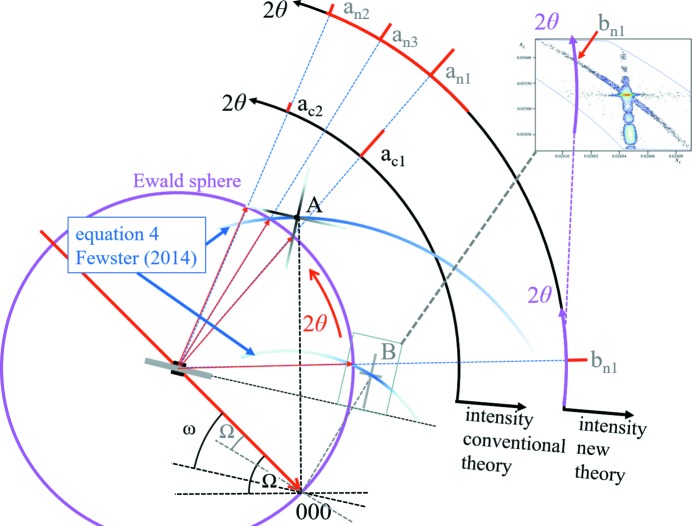
The interaction of different shape functions with the Ewald sphere. A gives rise to peaks a_c1_ and a_c2_ where the tails touch the Ewald sphere; this is the interpretation based on the conventional theory. In the new theory there is another term [equation (4), Fewster (2014 ▸)], so that three peaks appear a_n1_, a_n2_ and a_n3_ (a_n3_ is the enhancement peak) and there is also residual intensity associated with the whole of the shape function. The shape function given at B corresponds to the sample used in Fig. 8 ▸, *i.e.* for a crystal wafer with a truncation rod normal to the surface with a very short arm parallel to the surface. At this orientation the conventional theory predicts no peaks since no part of the shape function touches the Ewald sphere. The new theory predicts a peak at 2θ_B_ (b_n1_) for all orientations in Ω. The reciprocal-space map B can be compared with the measured data from Fig. 8 ▸ (inset) to show how a single extracted 2θ scan away from the Bragg condition forms enhanced intensity at 2θ_B_.

To explain the diffraction in the new theory compared with the conventional theory for a parallelepiped, consider Fig. 10 ▸ (shape function A), where its shape transform has been simplified to a cross with the tails diminishing in magnitude further from the reciprocal-lattice point. The conventional theory will reveal intensity where the shape transform intersects the Ewald sphere surface, resulting in two peaks. In the new theory the Ewald sphere surface has a thickness given by equation (4) of Fewster (2014 ▸). This results in intensity associated with all parts of the shape function and much of it will be very weak. The two peaks as in the conventional theory may well be the most dominant features; however, a strong feature like the maximum in the shape transform will also produce a peak, which may or may not be observed depending on the measurement conditions as in the examples above.

The example given in Fig. 8 ▸ has a shape transform like B in Fig. 10 ▸ and interacts with a different position on the Ewald sphere surface. The arc of intensity measured corresponds precisely to the prediction in the new theory. More details are given in the caption.

In the new theory, a very small crystal will have a very broad thickness profile for the surface of the Ewald sphere. This increases the observed intensity of features remote from the optimum position on the Ewald sphere surface; so, although the fringing could be touching the optimum position, the main peak in the shape transform can still dominate. This is exactly what is observed in the simulation from a perfect parallelepiped crystal in Fig. 2 ▸.

## 6. The difficulties with ‘conventional theory’   

Requiring crystals to be mosaic to suppress dynamical effects (Darwin, 1922 ▸) for the kinematical approximation to be applied in structure determination puts a big onus on all crystals. Is that reasonable? The number of crystals required to form a reliable polycrystalline diffraction pattern is greater than in a typical sample, in which case microdiffraction will not work; but it does, so what is going on? This did not go unnoticed by Alexander *et al.* (1948 ▸) who suggested crystals in a powder diffraction sample must be mosaic; but how small are they? De Wolff (1958 ▸) suggested that slack gearing in diffractometers may be the cause, but high-quality diffractometers of today would rule that out. Smith (1999 ▸) concluded that the data cannot be reliable even with the numbers of crystals used in Bragg–Brentano geometry. More recently, the data from XFELs show that there are reflections simultaneously observed in a snapshot from a single crystal, which should be a very rare event but is very common. This has led to a plethora of complex explanations to account for the data, *e.g.* Wojtas *et al.* (2017 ▸).

Each explanation is specific to the method by stretching the limits of conventional theory, which is in danger of becoming inconsistent with itself. The descriptions presented by the early workers in this field were valid explanations for their time, but perhaps they cannot be universally applied today. Suppose the fundamental theory is not the complete answer, then the results could be unreliable. Kuhn (2012 ▸) would view the conventional theory as a powerful paradigm needing a crisis, *e.g.* inexplicable results, to change it. Have we reached that stage yet? Or can the conventional theory still reveal reliable results? Popper (2002 ▸) suggested all theories are waiting to be disproved and therefore should be falsifiable. The assumptions in conventional theory have increased to accommodate these diverse experiments to prevent falsification. This situation is not favoured by the law of parsimony (Occam’s razor), which would prefer the theory with the fewest assumptions, because it is easier to falsify.

## 7. Conclusions   

The new theory explains the experimental results. There is, as far as I know, no alternative explanation within the confines of conventional theory. Those who can understand my description as well as the conventional theory should be able to compare these two approaches and make a judgement on which best describes their data. The new theory could be considered as defining a thickness profile for the Ewald sphere surface. In conventional theory this surface has no thickness, placing all the experimental interpretation on changing the shape of the reciprocal-lattice point, *e.g.* mosaic crystals. Shape effects cannot explain the results described above and therefore the conventional theory can only be an approximation. I consider my theory to be a better description of X-ray diffraction. The criticisms of my theory by Fraser & Wark are therefore based on an invalid argument.

## Footnotes

1 The peak 2θ_B_ values were determined with a ruler on the diffraction image and so could only be measured within a few % reliability, because of the peak breadth and the influence of the interference with the fringes, *e.g.* in the vicinity of the 120 peak position.

2 This divergence is based on dynamical theory, and also happens to be the same as the geometrically derived value based on the source size and a crystallite.

3 This is the measured width, whereas the intrinsic width is ∼0.0025°. An isolated 10 µm Si crystal within a polycrystalline sample (Fewster, 2014 ▸) gave a measured width of 0.002°, using a high-resolution diffractometer.

4 This tilt was determined by analysing the 004 reflection at opposite azimuths around the crystal surface normal.

5 I first considered this to be a problem with the diffractometer; however this is reproduced on different instruments based on different mechanical configurations and scanning arrangements.
